# Structural competency: Theorizing a new medical engagement with stigma and inequality

**DOI:** 10.1016/j.socscimed.2013.06.032

**Published:** 2014-02

**Authors:** Jonathan M. Metzl, Helena Hansen

**Affiliations:** a Center for Medicine, Health, and Society, Vanderbilt University, Nashville, TN, United States; b New York University, New York, NY, United States; c Nathan Kline Institute for Psychiatric Research, Orangeburg, NY, United States

**Keywords:** Stigma, Cultural competency, Medical education, Social determinates of health

## Abstract

This paper describes a shift in medical education away from pedagogic approaches to stigma and inequalities that emphasize cross-cultural understandings of individual patients, toward attention to forces that influence health outcomes at levels above individual interactions. It reviews existing structural approaches to stigma and health inequalities developed outside of medicine, and proposes changes to U.S. medical education that will infuse clinical training with a structural focus. The approach, termed “structural competency,” consists of training in five core competencies: 1) recognizing the structures that shape clinical interactions; 2) developing an extra-clinical language of structure; 3) rearticulating “cultural” formulations in structural terms; 4) observing and imagining structural interventions; and 5) developing structural humility. Examples are provided of structural health scholarship that should be adopted into medical didactic curricula, and of structural interventions that can provide participant-observation opportunities for clinical trainees. The paper ultimately argues that increasing recognition of the ways in which social and economic forces produce symptoms or methylate genes then needs to be better coupled with medical models for structural change.

## Introduction

A patient walks into a doctor’s office speaking a language that the doctor struggles to understand. The patient points to his chest while making pain gestures. Or mimics actions that suggest a seizure. Or fights to breathe. But the doctor is in her first week of residency, having just moved from rural Indiana to the Bronx, New York. And the patient grew up in low income housing and is on methadone maintenance. Or lives in a Hmong neighborhood where English is the third tongue. Or is an HIV-positive gay man who spends his life surrounded by a tight-knit community of orthodox Jews.

For much of the past two decades, “cultural competency” has been the rubric most often deployed in U.S. medical education for addressing the tensions of such moments of clinical encounter. Competency, in this formulation, implies the trained ability to identify cross-cultural expressions of illness and health, and to thus counteract the marginalization of patients by race, ethnicity, social class, religion, sexual orientation, or other markers of difference. Clinical professionals learn approaches to communication, diagnosis and treatment that take into account culturally specific sources of stigma, such as the stigma of mental health diagnoses among Asian immigrants, or the stigma of HIV and homosexuality in certain religious communities. Doctors train by analyzing vignettes that depict instances where “cultural” variables impact symptom presentations or attitudes about care. “*Mrs. Jones is an African American woman in her mid-60s who comes late to her office visit and refuses to take her blood pressure medication as prescribed*.” Or, “*You see a Mexican migrant who just received health counseling for Type II diabetes eating fried tortillas in the waiting room*.” Meanwhile, nurses develop “linguistic competencies” that teach them culturally sensitive, non-judgmental ways to build rapport with such patients. And pharmacists train in “communication skills” aimed to help build relationships when working in “multicultural settings” (American Association of Colleges of Pharmacy, 2006; Perez, 2008).

These are not insignificant developments. Cultural competency emerged during an era when U.S. medicine failed to acknowledge the importance of diversity issues (National Juneteenth Medical Commission). In the twenty years hence, it helped promote consideration of the impact of stigma and bias into treatment decisions. Yet the politics of the present moment challenge cultural competency’s basic premise: that having a culturally sensitive clinician reduces patients’ overall experience of stigma or improves health outcomes. Increasingly, we hear that low-income African Americans are unable to comply with doctors’ orders to take their medications with food, not because they harbor cultural mistrust of the medical establishment, but because they live in food deserts with no access to grocery stores. Or, that Central American immigrants who are at risk for Type-II Diabetes refuse to exercise, not because they are uneducated about the benefits of weight reduction, but because their neighborhoods have no gyms or sidewalks or parks. Or, that small numbers of opulent white Americans pay for their healthcare out of pocket, not because they do not qualify for coverage, but because the tax breaks and advantages they receive allow them to pay cash for office visits with elite practitioners who do not accept insurance. Or even that doctors overlook “cultural” variables, not because they are insensitive, but because they work in clinics with inadequate resources, and dwindling community support. These and other encounters suggest how the clinical presentations of persons at both ends of the economic spectrum are shaped by “cultural” variables, and also by the economic and political conditions that produce and racialize inequalities in health in the first place. And, that stigma and cultural conflict in health-care settings needs be understood as the sequellae of a host of financial, legal, governmental, and ultimately ethical decisions with which medicine must engage politically if it wishes to help its patients clinically.

This paper tracks an evolving discourse that redefines cultural competency in structural terms. We theorize a five-step conceptual model meant to promote awareness of forces that influence health outcomes at levels above individual interactions. We argue that, if stigmas are not primarily produced in individual encounters but are enacted there due to structural causes, it then follows that clinical training must shift its gaze from an exclusive focus on the individual encounter to include the organization of institutions and policies, as well as of neighborhoods and cities, if clinicians are to impact stigma-related health inequalities.

As this special issue attests, public health, social science, and critical race studies scholars have, over the last decade, begun to locate stigma, not just in the attitudes of individual persons, but in the actions of institutions, markets, and health care delivery systems (Bonilla-Silva, 2003; Hatzenbuehler & Link, 2014). This literature importantly reveals how stigma in clinical encounters needs be addressed in the institutions and social conditions that produce the markers of exclusion that we call stigma, as well as in on-the-ground encounters. Similar sensibilities now suffuse a number of interventions that address the material realities of illness and health. These interventions have, to this point, been disparate and disciplinary, and thus largely developed outside of clinical practice. For instance, global-health students at Harvard learn to think about “sickness,” diagnosis, and treatment in relation to food and medication distribution networks (Farmer, Nizeye, Stulac, & Keshavjee, 2006). Masters students at the Michigan College of Architecture and Urban Design form the first cohort of a new program in Design/Health, train to build city environments that promote health (Taubman College). And sociologists learn to observe the interplay of social structures and “neighborhood effects” (Sampson, 2012). These and other initiatives suggest possibilities for a major shift in the objects of clinical intervention assumed by cultural competency training, and in the broader outcomes sought by considering the impact of “culture” on clinical interactions.

We cull generalizable principles from a number of medical and extra-medical literatures to propose a new paradigm for medical education, *structural competency* (Metzl 2010; structuralcompetency.com). Central to our intervention is the belief that, just as stigma in clinical encounters must be addressed structurally, so too must inequalities in health be conceptualized in relation to the institutions and social conditions that determine health related resources. We contend that medical education needs to more systematically train health-care professionals to think about how such variables as race, class, gender, and ethnicity are shaped both by the interactions of two persons in a room, and by the larger structural contexts in which their interactions take place. And, that as such, clinicians require skills that help them treat persons that come to clinics as patients, and at the same time recognize how social and economic determinants, biases, inequities, and blind spots shape health and illness long before doctors or patients enter examination rooms.

In 1968, the civil-rights activist Stokely Carmichael famously assailed forms of racial bias embedded, not in actions or beliefs of individuals, but in the functions of social structures and institutions. “I don’t deal with the individual,” he said. “I think it’s a cop out when people talk about the individual.” Instead, speaking to a group of mental-health practitioners, Carmichael protested the silent racism of “established and respected forces in the society” that functioned above the level of individual perceptions or intentions, and that worked to maintain the status quo through such structures as zoning laws, economics, schools, and courts. Institutionalized racism, he argued, “is less overt, far more subtle, less identifiable in terms of specific individuals committing the acts, but is no less destructive of human life” (Carmichael, 2003: 151).

Attention to structure as an organizing principle in medical education seems particularly important at the present moment because the forces Carmichael described have become ever-more destructive to human life. Evidence also suggests that inattention to these forces has caused a crisis of confidence for which American medical education is ill-prepared.

On the one hand, US physicians have never known more about the ways in which the pathologies of social systems impact the material realities of their patient’s lives. Epigenetics research demonstrates, at the level of gene methylation, how high-stress, resource-poor environments can produce risk factors for disease that last for generations (Johnstone & Baylin, 2010). Meanwhile, nueuroscientists show neuronal linkages between social exclusion, poverty, hampered brain development, and mental disorders (Buwaldaa et al. 2005; Evans, 2009). And economists prove that low income persons can reduce their rates of obesity, diabetes, and major depression by moving to safer, more affluent neighborhoods (Judwig, 2011). These are but a few examples of the types of research that doctors can now access—at a level of microscopic and macroscopic precision unimaginable in Carmichael’s time—to understand how diseased or impoverished economic infrastructures can lead to diseased or impoverished, or imbalanced bodies or minds. And, how locating race-based symptoms on the bodies of marginalized or mainstream persons risks turning a blind eye to the racialized, stratified economies in which marginalized and mainstreamed bodies live, work, and attempt to survive.

On the other hand, many of these physicians work in a country that has never invested less in infrastructure, or done less to correct fatal and fatalizing inequities—even in the aftermath of the Affordable Care Act. Bridges, roads, clinics, and public transportation and food distribution programs decay in many US urban settings, along with the social programs that sustained them (Davey, 2011). Some locales prosper, while many others face a state that urban planners define as “infrastructure failure.” As **U.S.** Housing and Urban Development Secretary Shaun Donovan recently put it, “you can predict the life expectancy of a child by the zip code in which they grow up” (Bostic & Lavizzo-Mourey, 2011).

This divergence, between knowing a lot about the health effects of wealth imbalances and doing little to address them, puts US medicine in a particular bind. Its practitioners ostensibly want to help the persons who come before them in times of need. Yet when “social” issues are at play, these practitioners often know not what to do about it. Fully 85% of primary care providers and pediatricians polled in a recent Robert Wood Johnson survey agreed with the statement that “unmet social needs are leading directly to worse health for all Americans” while at the same time voicing concern that they did not “feel confident in their capacity to meet their patients’ social needs,” and that their failure to do so “impedes their ability to provide care” (Harris Interactive, 2011). Meanwhile, increasing numbers of physicians cite structural factors, such as restrictive insurance policies or lack of time with patients, as reasons to leave clinical practice (Pathman et al., 2002).

Many complex reasons underlie this frustration with addressing social issues. Vast wealth disparities undoubtedly foment feelings of learned helplessness, as gaps between rich and poor or health and illness become mortared into mortal logics of common sense. But perhaps one explanation for the insecurity rests in Carmichael’s argument: when structural violence–systemic institutional stigmatization and marginalization–is at issue, we train doctors to listen to individualized stories, not to structural ones. For instance, methods such as cultural competency or narrative analysis teach doctors to better listen to the “cross-cultural” aspects of the stories that their patients tell at moments of clinical encounter and within the context of doctor-patient interactions. While such approaches enhance clinical dialogue in vital ways, they do little to address the complex relationships between clinical symptoms and social, political, and economic systems. We thus argue that medical education needs to more broadly engage with knowledges and methods beyond its traditional purview if it wishes to train its practitioners to effectively address the pressing health issues of our time. And, ultimately, that increasing recognition of the ways in which social and economic forces produce symptoms or methylate genes then needs to be better coupled with medical models for structural change.

## Structural competency

We define structural competency as the trained ability to discern how a host of issues defined clinically as symptoms, attitudes, or diseases (e.g., depression, hypertension, obesity, smoking, medication “non-compliance,” trauma, psychosis) also represent the downstream implications of a number of upstream decisions about such matters as health care and food delivery systems, zoning laws, urban and rural infrastructures, medicalization, or even about the very definitions of illness and health.

Structure is of course a term with a complex theoretical past, from Marx to Giddens to Levi-Straus. In our formulation, structure implies the buildings, energy networks, water, sewage, food and waste distribution systems, highways, airline, train and road complexes, and electronic communications systems that are concomitantly local and global, and that function as central arteries in some locales and as sclerotic corollaries in others. Structure also demarcates the oft-invisible diagnostic and bureaucratic frameworks that surround biomedical interactions, and that potentially shape the contents there within. And, structure connotes assumptions embedded in language and attitude that serve as rhetorical social conduits for some groups of persons, and as barriers to others.

Of course, attention to forms of structure marked earlier attempts to impact the health implications of wealth imbalances or treat the embodied effects of social and economic problems. In the 1960s and 1970s, nurses, physicians, and social workers in the Community Mental Health Movement created a series of social support networks in their attempts to shift mental illness treatments from hospitals to communities (Grob, 1994). Political protest groups linked health care delivery systems to broader struggles for social justice, such as the Black Panther Party’s free clinics that called attention to medical discrimination (Metzl, 2010; Nelson, 2011).

As we detail throughout this paper, differing notions of structure also figure prominently in a number of present-day discourses that help explain attitudes about, and stigmatizations of, illness and health. Stigma researchers highlight ways in which stigma is produced by structural or institutional forces, such as unequal access to treatment, unfair tax codes, or discriminatory laws. Meanwhile, social scientists and humanities scholars add important conceptualizations of structure as a system that produces and reproduces the social world, and that is thus deeply linked to culture because it provides the system of values affixed to bodies and diseases. And political and public-health activists use structures of oppression, such as racism or debt, to address seemingly biological conditions of morbidity and mortality. Calling on these and other literatures, structural competency seeks to promote skills, not so much for replacing awareness of “culture” in medical settings, but for recognizing how “culture” and “structure” are mutually coimplicated in producing stigma and inequality.

Competency, meanwhile, does not imply mastery of these protean forces within the context of already overbooked schedules or curricula. Medical education has of late developed a potential over-competency syndrome, claiming expertise over a range of highly complex topics that have eluded humanities and social science scholars for years—recent initiatives call for doctors to develop “gender and sex competencies” and “religious competency,” as but a few examples. We find common ground in the belief that conceptualizing and intervening into abstract social formations is a skill that requires study and practice over time. And, that the competency that results from such efforts helps clinicians develop, not the hubris of mastery, but the humility to recognize the complexity of the structural constraints that patients and doctors operate within (Tervalon & Murray-Garcia, 1998).

In what follows, we call on the expansive interdisciplinary literature on structure to illustrate five tenets of our theoretical approach.

## Core structural competencies

Five intersecting skill-sets shape the paradigm of structural competency proposed here:

### 1. Recognizing the structures that shape clinical interactions

The first component of structural competency promotes recognition of how economic, physical, and socio-political forces impact medical decisions. For instance, a traditional cultural competency approach might address the vignette cited above—“*Mrs. Jones is an African American woman in her mid-60s who comes late to her office visit and refuses to take her blood pressure medication as prescribed*” —through a series of questions about Mrs. Jones’s background or her attitudes about medications. Taking nothing away from the relevance of these factors, a structural approach would ask students to narrate case studies that uncover how frames and constraints from beyond the exam-room walls might impact the case. Students might be asked to analyze the trope of time pressure inherent in the vignette by researching how insurance, hospital, or healthcare administration policies dictate the amount of time that the doctor can spend with Mrs. Jones—and to consider how the amount of time that the doctor spends with Mrs. Jones influences the content of the conversation that they may or may not have.

Students might also be asked to narrate Mrs. Jones’s medications, not just based on whether they are clinically indicated, but on their pharacoeconomics. Does Mrs. Jones receive brand-name or generic prescriptions? What policies impact the prices for these medications? Where does she fill the prescriptions? Or, students might narrate the case through attention to the function of diagnostic structures. Here an instructor might ask, what purpose surrounds Mrs. Jones’s diagnosis of hypertension, and what larger function does the diagnosis serve? Where else, beyond the clinical chart, will the diagnosis appear, and to what effect?

Instructors might then asked students unpack these issues by combining research into particular hospital, insurance, or agency policies with reference to the extensive literatures that address each structural theme. Dugdale, Epstein, and Pantilat (2001) and Saunders (2008), for instance, provide important jumping off points for discussing time valuations in clinical settings. Angell and Relman (2002) and Finkelstein and Temin (2008) analyze the governmental policies that contribute to pricing medicines and controlling competition for generic drugs, while Hansen and Roberts (2012) demonstrate the legal, marketing, and regulatory strategies used to promote prescription drugs to different “ethnic” populations. And Metzl (2010) critiques the “culture of diagnosis” and analyzes the racialized implications of diagnostic frames.

Of course, clinicians often understandably feel that issues such as time or drug pricing are matters over which they have little control. Yet cognizance of the mechanisms that produce such emotions seems a productive first step in addressing them. The purpose of this first component of structural competency is thus to introduce constructive ways of discussing how upstream decisions about resources, and the political economy of healthcare in the U.S., impacts clinicians as well as patients. And, how attention to these factors provides a deeper understanding of the tensions that might arise between the doctor and Mrs. Jones.

### 2. Developing an extra-clinical language of structure

The second component of structural competency shifts emphasis beyond hospitals or clinics by imparting fluency in disciplinary and interdisciplinary understandings of structure as they pertain to illness and health in community settings. To be sure, attention to infrastructure suffuses such public-health and biomedical literatures as social determinates of health, health disparities, or epigenetics. Researchers show, with emerging specificity, how resource-poor environments elicit a range of physiological and cellular adaptive responses that lead to chronic diseases such as type-two diabetes and coronary heart disease, for instance (Ozanne & Constância, 2007).

While research increasingly demonstrates the impact of social environments on metabolisms or genetics, concepts of actual social structures and social forces lag behind. Studies that demonstrate the physiologic effects of racism on cortisol levels contain little discussion of the nature of racism itself, or of the social hierarchies that promote its ill effects (Tull, Sheu, Butler, & Cornelious, 2005). Meanwhile, research that so effectively illuminates ways in which decayed infrastructures lead to specific bodily illness often provide relatively less detail about structural level interventions for complex social problems, beyond requisite calls for policy changes or for increased investment in poor areas. Biomedicine thus conveys highly advanced knowledge of the biological impacts of lived environments alongside relatively undertheorized analyses of the environments themselves. The result—as the survey cited above (Harris Interactive, 2011) suggests—flattens medical abilities to discuss the “social” aspects of social determinates. Social, in this biomedical frame, becomes a monolithic or immutable force that functions beyond the reach of medical imagination or expertise.

Structural competency seeks to expand medical educational approaches to social realms by infusing into medical canon scholarship on the hierarchies, economies, and networks through which health and illness are produced and maintained. Medical anthropologists, for example, study socially structured patterns of disease across population groups and economies in ways that point to structural agendas for political and economic change. Farmer (2001: 79) argues that epidemics such as tuberculosis and HIV are the result of “structural violence”: “neither culture nor pure individual will is at fault; rather, historically given (and often economically driven) processes and forces conspire to constrain individual agency.” Parker and Aggleton (2003) further link the concept of structural causation to the concept of stigma. They point out that individual experiences of stigma are inextricably linked to the political and economic systems that create it. And Quesada, Adams, Bourgois, and others (Adams, Kaufman, VanHattum, & Moody, 2011; Quesada, Hart, & Bourgois, 2011) describe a “structural vulnerability” to the forces that constrain decision making, frame choices, and limit life options and therefore health for the disadvantaged.

Medical sociology contributes important analyses of the relationships between notions of structure and agency as they pertain to illness and health. Complicating structural vulnerability’s contention that structure constrains agency, sociologists study ways in which persons navigate resource-poor environments by making volitional choices that impact morbidities and mortalities. Bourdieu’s (1977) notions of the ways in which external class structures are internalized as personal bodily and mental habits often serves as the jumping off point for this literature’s engagement with structure. Sociologists also incorporate notions of agency (Sewell, 1992) that argue that social actors are knowledgeable and capable of acting in creative ways that can ultimately transform structure from within. For instance, Rose (2011) studies residents of impoverished Detroit neighborhoods to chronicle food “acquisition strategies” through which residents band together to form shopping cooperatives and travel pools, thus gaining some measure of control over their diets and their health choices.

Medical sociologists also demonstrate how health disparities reflect systems of privilege, and how understanding the health of persons at the bottom of the economic structure requires understanding the patterns of resource utilization deployed by persons at the top. Williams (1996), Schulz et al. (2000), and McDonough (2000) demonstrate how specific structural forces such as neighborhood effects and income dynamics benefit the health of certain persons while at the same time having disastrous consequences for the mortality of others.

Architecture, urban planning, and geography approach structure through grid, mortar, and steel. Grahm’s notion of “infrastructure failure” catalogues the impact of decaying energy, water, sewerage, transport, trade, finance, and communication infrastructures on public health, while Richard Little studies how disruptions in urban infrastructure networks “cascade” rapidly and unpredictably through other infrastructures, impeding emergency medical care networks (Grahm, 2009).

As this special issue details, multidisciplinary scholars describe how structural stigma operates to produce a wide range of observations, from the association of the mortality of sexual minorities with state level policies on same sex relationships (Hatzenbuehler et al., 2014), to the ways that institutional racism operates in medical education to perpetual health care disparities (Feagin & Bennefield, 2014), to the relationship of welfare reform to the rise in disability applications and identification of the poor as diseased (Hansen et al., this issue).

Meanwhile, economists such as Sen (1999) theorize ways in which structural inequalities built into financial systems work to deny basic human needs and ultimately limit life expectancies. Historians such as Wailoo (2001) analyze how structures of medical systems shape illness categories and notions of health “disparities” at different moments in time. And disability studies scholars such as Kirkland (2008) show how structural assumptions about “healthy” body size and function implicitly stigmatize persons who fall outside of aesthetic norms.

Pedagogic engagement with these complex notions of structure should not require medical students and residents to memorize voluminous and complex literatures in their limited time. Instead, introduction to interdisciplinary literatures of structure helps these clinicians begin to articulate how the persons, diseases, and attitudes that appear before them can be better understood as the product of social structures interacting with biologies. And, that as such, the seemingly straightforward case of Mrs. Jones is even more complex than it may once have seemed.

### 3. Rearticulating “cultural” presentations in structural terms

The third component of structural competency promotes the ability to reformulate “cultural” clinical presentations with terms and concepts from the interdisciplinary literature of structure detailed above. Again, the aim here is not to “replace” culture or to eschew discussions about cultural values between patients and doctors. At the same time, evidence suggests that the notion of culture deployed in clinical settings emphasizes familiarity with the values of different class groupings or ethnic communities, while potentially effacing recognition of the deeper ways in which complex cultural structures produce inequalities and create barriers to inclusion (Carpenter-Song, Nordquest, & Longhofer, 2007).

For instance, Hansen’s and Dugan’s (2013) interviews with faculty from twenty U.S. medical schools revealed the limitations of the language and logic of the term “cultural” to describe issues that would be more adequately described as structural determinates of health. Although most respondents in their study expressed motivation for teaching about “health disparities,” the majority of faculty members interviewed did not explicitly examine institutions or policies that might account for such disparities, but rather, spoke of “culture” primarily in terms of ethnic identity. Meanwhile, Willen, Bullon, and Good (2010) and Hannah and Carpenter-Song (2013) highlight the potential frustrations engendered by asking doctors to master cultural competencies that are at times foreign or uncomfortable for them.

The language of structure provides a more constructive alternative, because it allows medical classrooms to emphasize how “cultural” barriers arise when structural forces manifest themselves in patterns of interpersonal communication and institutional practices. Continuing the reading of the vignette above, questions here might include, what social, infrastructural, or economic factors might enable or delay Mrs. Jones’s transit to the appointment? What community based factors might impact, positively or negatively, the need for the appointment in the first place?

Students might then be asked to narrate a second, patient-centered reading of the case that emphasizes structural barriers to, and stigmatizations of, health. Here they might call on Rose (2011) to ask Mrs. Jones about her “strategies” for getting to the clinic. Or, they might use McDonough, Duncan, Williams, and House (2000) to research how infrastructures pertaining to transportation, food, or medical care reflect “income dynamics” of urban space. They might also use the excellent “structural vulnerability” checklist produced by Quesada et al. (2011) to talk about the relationships between the infrastructure that surrounds Mrs. Jones’s neighborhood and health of its inhabitants. Or, they might deploy Sen (1999), Farmer et al. (2006), or Hatzenbuehler (in this issue) to discuss the relationships among economies, biases, and attitudes that Mrs. Jones’s illness illustrates.

Reading the case as such might require medical instructors and students to call on the expertise, not just of social determinants of health researchers, but of colleagues from arts and sciences domains—this is in part the point. Such collaborations can then impart fuller understanding of the structural forces that patients must traverse in order to receive health-care, and the invisible barriers that stand in the way. Over time, medical education then begins to develop a richer vocabulary for rendering structural mechanisms of stigma and marginalization visible, while at the same time shifting diagnostic focus from the “culture” of individual patients to the cultures of privilege and oppression that structures, like human constructions, represent.

### 4. Observing and imagining structural intervention

The fourth component of structural competency seeks to impart recognition that structures that shape health and illness are neither timeless nor immutable, but instead reflect specific financial, legislative, or indeed cultural decisions made at particular moments in time. And, that as such, these structures are subject to various forms of intervention. Here, the question might be, given that we have now identified how structures impact the experiences of the doctor and Mrs. Jones, what can we do to intervene? This component thus moves trainees toward real world application by asking students to analyze structural interventions and then propose interventions that address health infrastructures.

This aspect of competency allows for multiple forms of observation. A natural entry point might be the deployment of historical observation—oral histories, archival analyses, literature searches—to analyze earlier medical attempts to address social justice issues. For instance, in the 1960s, activist-physician Jack Gieger founded one of the nation’s first community health centers in the Mississippi delta. Gieger famously wrote prescriptions for food—-stipulating quantities of milk, vegetables, meat, and fruit that could be “filled” at grocery stores, along with instructions to send the bills to the health center” (Bornstein, 2011).

Or, students might observe politically, by studying activist organizations that protest structural health issues. For instance, the OccupyWall Street offshoot group Strikedebt (http://strikedebt.org/ lifeordebt) organizes “life or debt” protests that highlight the crushing impact of medical bankruptcies.

Or, students might ethnographically observe any one of a number of present-day organizations or clinics in both wealthy and poor neighborhoods that address the medical implications of social issues. An approach here might ask trainees to complete clinical ethnographies (Kleinman & Benson, 2006) of community-based interventions, taking field notes based on participant observation in sites of structural interventions, and interviewing staff and clients about the way the intervention impacts of the daily lives of participants. For instance, students might observe how Mindy Fullilove treats cities that have been “fractured and wounded” by racially segregating urban renewal and redlining policies, rather than individual patients. Fulliove works with community based organizations, urban planners, and architects to promote “nine elements of restoration” —including creating healthy spaces for use by all city residents (Fullilove, 2013). Conversely, trainees might chose to study the structures of health surrounding boutique, cashonly medical clinics that function above communal infrastructures of health insurance.

A structural competency curriculum might also assign teams of medical trainees to design their own structural interventions. Lest students think that implementation is not possible within the rigors of pre-professional training, an instructor might point to any number of student-initiated or student-run programs. For instance, Health Leads, an organization founded by Rebecca Onie while an undergraduate at Harvard University, provides resource desks in waiting rooms of urban health centers. At these sites, doctors “prescribe” a wide range of basic resources, like food assistance or heating fuel subsidies, which Health Leads’ volunteers “fill” (Health Leads). Similarly, medical students in Tennessee, observing that minority and low income patients failed to comply with instructions to take their medications after meals because they had to travel over two hours to reach the nearest grocery stores, created a social enterprise program called Nashville Mobile Market that hires refrigerated food trucks to deliver food and other items to impoverished areas (Nashville Mobile Market).

Advanced trainees might also intern in long-term collaborative health interventions that seek to impart national or global policy change. Partners in Health, a well-known example, began by with clinical programs to provide state-of-the-art medication in global regions that were structurally vulnerable to epidemics, and then lobbied for change of the global policies which had displaced populations and concentrated poverty in the first place (Farmer, 2003). In the U.S. context, researchers at the Substance Abuse and Mental Health Services Administration work at the Federal level to promote the concept of “recovery” as the goal of mental health treatment, in which mental health service users actively decide on their desired treatment outcomes (Ademola,Whitley, & Kirmayer, 2012; Jacobson & Greenly, 2001). This patient-centered approach complements a parallel movement in health research called community-based participatory research (Wells & Jones, 2009). Recovery and community-based participatory involvement also mark interventions byMary Jane Alexander and Kim Hopper (Center to Study Recovery in Social Contexts, 2012; Hopper, 2007), Harold Freeman (hpfreemanpni.org), and Benjamin Springgate and colleagues (2011).

Obviously, observing these interventions require skills that medical students and residents cannot master while on brief electives or rotations. They key is to build exposure to such projects into the curriculum for trainees, either on-site through internships or fellowships, or remotely via classroom presentations or structural competency grand rounds. Questions asked in written assignments or in-class or in-clinic discussions might then include: what “problems” do organizations or interventions aim to address? Which notions of structure from parts 1, 2, and 3 above—e.g., medical, anthropological, sociological, historical—are most helpful when identifying problems and conceptualizing solutions? What are the barriers to, and benchmarks of, treatment or success over time? What types of interventions can you imagine or enact that might also address structural health issues?

### 5. Developing structural humility

The final component of structural competency is the trained ability to recognize the limitations of structural competency. Here, students demonstrate a critical awareness of medical education’s realistic goals and endpoints. The term humility usefully comes from medical educators (Hunt, 2001) who voice critique of cultural competency through the concept, developed by the philosopher Emmanuel Levinas, that the Other always lies beyond the comprehension of the self. Writing of cross-cultural clinical encounters, DasGupta (2008) asserts that doctors who assume that their reading of a patient’s narrative is the “definitive interpretation” risk closing themselves off to awareness of the patient’s “valuable nuances and particularities.”

So too, the forces of structure defined above rarely sit still for diagnosis, and indeed the very premise of medical mastery over complex, evolving economic and structural issues—or indeed over the material conditions of Mrs Jones’s life—often *a priori* acknowledge the limitations of medical expertise. For instance, while the rhetoric of “prescribing” food or services seems on its face to reinforce medical authority, the skills required for implementation call on networks and skill-sets for which clinicians are rarely trained. Meanwhile, moves to “medicalize” architecture and urban planning have come under critique for promoting particular classist notions of health, notions that become apparent when low income community members are included in the process of urban planning (Borasi & Zardini, 2012).

Awareness of this complexity is productive, as long as practitioners of structural competency recognize that the skills they develop are the beginning points of conversations rather than endpoints. And, that in these conversations, clinicians are at once speakers and listeners, leaders and collaborators, experts and benighted.

## Conclusion

Addressing stigma and inequality in clinical settings requires that clinicians attend to the social structures that shape and enable stigma’s underlying assumptions. However, these structures are frequently rendered invisible in medical education. Promoting awareness of structural forces serves as a first step toward promoting recognition of the web of interpersonal networks, environmental factors and political/socioeconomic forces that surround clinical encounters and of better understanding the conversations that take place there within. Starting with medical education is a modest attempt to begin to promote new forms of coalition in which knowledge about diseases and bodies combines with expert analysis of social systems in ways that might, over time, might help put notions of structural stigma at the center of conceptualizations of illness and health.

It is of course the case that some of the interventions we describe above, and many that we do not, already appear in certain medical-school curricula. For instance, Albert Einstein medical college in New York promotes a “research-based health activism program” that combines clinical research and epidemiology with grass-roots advocacy in an attempt to train future doctors to “advocate for public health, social justice, and health equality” (Albert Einstein College of Medicine). Meanwhile, two physicians at the University of Michigan, Kumagai and Lypson (2009), developed a medical school curriculum aimed at developing “critical consciousness” —a skill that “places medicine in a social, cultural, and historical context and which is coupled with an active recognition of societal problems and a search for appropriate solutions.” And the Accreditation Council for Graduate Medical Education boasts an impressive list of “healthcare disparities competencies” for residents (abp.org).

Structural competency is an attempt to broaden these types of skills into more expansive realms of education and practice. We recognize that a call to competency risks promoting checklists of facts for didactic instruction, rather than preparation for careerlong engagement with learning and acting on the structural determinants of stigma and health across disciplines and communities. At the same time, competency emphasizes ability, and the promise of remediation. Competency also indicates a set of proclivities that are essential to the role of health care provider, including the duty of providers to cultivate in themselves, and the duty of medical educators to impart to trainees.

What, then, would a structurally competent clinician look like? We hold that this clinician would possess skills of differential diagnosis that would enable her or him to entertain multiple interpretations for scenarios whose tensions are, in the heat of the moment, too-often reduced to explanatory models based in cultures, ethnicities, or other urgencies of here-and-now clinical encounters. Thus, the aforementioned scenario might produce interpretations and questions in excess of those based in doctorpatient rapport or cultural mistrust of physicians. “Mrs. Jones is an African American woman in her mid-60s who refuses to take her blood pressure medication.” Again, a structurally competent student might also ask, where and how does Mrs. Jones get her medications? Or, where is the nearest bus stop to her home? Or, what networks or alliances or neighborhood effects might enable or block the path from prescription to payment to ingestion to mistrust? Or, what social structures and structural stigmatizations might the otherwise beneficial medications represent? Or, what other ways, besides interpersonal reassurance, might help rectify the inherent tensions of the encounter before Mrs. Jones comes to the clinic in the first place?

Ultimately, this clinician might expertly recognize that individual competency is a false-front, in as much as the expert gaze of the physician who seeks to deliver structurally competent care works best if situated within groups or networks that are also concerned with the health of Mrs. Jones. And as such, this clinician might join medical calls for structurally competent institutions, agencies, networks, and politicians, even as she or he participates in training modules that are all-too-often geared toward sole practitioners.

In no way does this approach obviate the importance of interpersonal communication in clinical interactions, and particularly interactions that are often more challenging when clinicians and patients attempt to traverse cultural, language, economic, or other forms of difference. At the same time, medicine has for too long located the clinical encounter as the primary site of politics. Getting the diagnosis *right* is of course one of the more important medical skills. But overlooking the ways that individuals are constrained and shaped by structural factors leaves clinicians unprepared for the types of group-level conversations that will become increasingly prevalent as the U.S. continues to addresses the relationships between medical expenditures and our growing GDP.

Our call for structural competency is ultimately a call for a language can help medicine combat the learned helplessness that, as the RWJ study above suggests, often accompanies structural health issues. And, that allows medical education to participate more fully in micro- and macro-level negotiations about structural issues inways that protect the welfare of medicine *writ large*, while at the same time championing the interests of the persons, neighborhoods, and infrastructures to which medicine owes its mission and its own wellbeing.
